# Case of Hæmorrhage from a Branch of the Anterior Palatine Artery

**Published:** 1863-03

**Authors:** H. T. Kempton


Case of Hæmorrhage from a Branch of the Anterior Palatine Artery—*By H. T. Kempton*, F. L. S. M. M——, a female, aged 35, consulted me on the 10th of May last, having the night before awoke in great alarm, owing to a quantity of coagulated blood getting into the throat and producing a sense of suffocation. On examing the mouth, I first thought the bleeding proceeded from the gums surrounding some stumps in the upper jaw, over which a plate of gold had been fitted with artificial teeth. On inquiry, I found the teeth had not been removed since I placed them in the mouth just twelve months prior to this visit. Consequently, on removing them, I found the gums and that part of the palate covered by the plate very much swollen and inflamed; no bleeding occurred on the immediate removal of the plate, but after rinsing the mouth with warm water, a quantity of arterial blood suddenly escaped into the mouth, which issued in jets, and must have come from a branch of the anterior palatine artery, behind the incisor teeth, just in the median line of the mouth, and in the centre of the part covered by the gold plate. I immediately applied a compress of lint steeped in spirit of wine, and kept up pressure by replacing the plate with the artificial teeth, which at once arrested the hæmorrhage. I requested the patient to keep herself perfectly quiet, and on retiring to rest to have the head raised considerably above the body, and to call upon me early the next morning; but if in the interim bleeding shall return, to call in medical aid. She, however, came the next morning and informed me that only a very slight oozing had occurred since her visit the day before; but on removing the compress a small stream came from the artery; I again applied a dossil of lint steeped in spirit of wine, and secured it as before. The patient called the following morning, when I removed the compress, and as no bleeding occurred on rinsing the mouth with tepid water, I replaced the teeth without any further application. On visiting me a few days afterwards, she stated that since her last visit no unfavorable symptoms had returned.

In the above case there are two points worthy of the notice of the junior members of our profession. First, the importance of patients having artificial teeth frequently removed, in order that they may be kept perfectly clean, as independently of small particles of food accumulating from time to time, and imparting a most offensive odour to the breath, they also retain the epithelium, which in time becomes destroyed, and leaves a highly sensitive deep red surface, and if allowed to continue, is likely to lead to more serious results than in the above case. Secondly, the simplicity of the application. Every dentist has spirits of wine at hand, therefore no time need be lost. Should it not suffice to arrest hæmorrhage, it will afford time for more coagulants to be obtained. If I had not succeeded with the spirits of wine, I should have used creosote, having seen it applied by my friend and colleague, Mr. Hulme, with the most perfect success, in arresting severe hæmorrhage after the extraction of a large upper molar tooth.

17 Cavendish place.—*London Dental Review.*

